# Transcultural adaptation and assessment of psychometric properties of the Spanish version of the Scale for the Evaluation of Staff-Patient Interactions in Progress Notes

**DOI:** 10.1371/journal.pone.0281832

**Published:** 2023-03-28

**Authors:** Alonso Pérez-Toribio, Antonio R. Moreno-Poyato, María Teresa Lluch-Canut, Michael Nash, Montserrat Cañabate-Ros, Kjellaug K. Myklebust, Stål Bjørkly, Montserrat Puig-Llobet, Juan F. Roldán-Merino

**Affiliations:** 1 Unitat de Salut Mental de l’Hospitalet, Servei d’Atenció Primària Delta de Llobregat, Direcció d’Atenció Primària Costa de Ponent, Institut Català de la Salut, Barcelona, Spain; 2 Department of Public Health, Mental Health and Maternal and Child Health Nursing, Nursing School, Universitat de Barcelona, L´Hospitalet de Llobregat, Spain; 3 School of Nursing and Midwifery, Trinity College Dublin, Dublin, Ireland; 4 TXP Research Groups, Universidad Cardenal Herrera-CEU, Valencia, Spain; 5 Hospital Clínico Universitario, Valencia, Spain; 6 Faculty of Health Sciences and Social Care, Molde University College, Molde, Norway; 7 Centre for Forensic Psychiatry, University Hospital, Oslo, Norway; 8 Campus Docent Sant Joan de Déu Fundació Privada, School of Nursing, University of Barcelona, Barcelona, Spain; Universidad de Zaragoza, SPAIN

## Abstract

**Purpose:**

To adapt the Scale for the Evaluation of Staff Patient Interactions in Progress Notes to Spanish and to test the psychometric properties.

**Design and methods:**

The study was conducted in two phases: (1) Adaptation of the instrument to Spanish following the Standards for Educational and Psychological Testing. (2) Psychometric study in a sample of mental health nurses.

**Findings:**

The Cronbach’s alpha values were 0.97 for the total scale and 0.83 to 0.81 for each dimension. The inter-rater reliability values were between 0.94 and 0.97.

**Practice implications:**

The scale is a reliable tool for assessing nurses’ clinical notes in relation to the quality of nurse-patient interactions.

## Introduction

In mental health nursing practice, the therapeutic relationship between the nurse and the patient is of special importance [1]. Therefore, it is essential that the quality and extent of nurse-patient interactions that occur in the context of the relationship are properly described in the nursing documentation [2].

The therapeutic relationship is known to be the cornerstone of mental health nursing [3, 4]. This is characterized by the interpersonal relationship that is established between the nurse and the patient, a relationship based on trust between the two parties, and focused on therapeutic assistance [5]. Through the therapeutic relationship, the nurse helps the patient to develop and work individually towards their wellbeing, thus empowering the patient in their recovery process [6]. Establishing an appropriate therapeutic relationship is associated with better health outcomes for patients, improving the effectiveness of interventions in inpatient mental health care and enhancing the well-being and experience of patients as well as nurses [7, 8]. For this reason, it is important to make nurses aware of the importance of maintaining a quality relationship with patients [9]. A relationship based on a solid bond that enables understanding of the patient’s needs and helps the relationship process to reach an agreement on the objectives and interventions to be carried out with the patient [10].

However, it is important to note that any nurse-patient interaction can be therapeutic, while at the same time it has the potential to be nontherapeutic if conducted incorrectly [11]. The literature identifies the most important barriers to establishing the therapeutic relationship as perceived by nurses: a) excessive administrative tasks that limit the time available for other activities [12], b) the lack of private space [5], c) lack of leadership and support from supervisors [13]. In addition, the biomedical model also constitutes a barrier to the therapeutic relationship [14]. This model conditions the language used in mental health nursing, a particularly important language that should be of great use for building and maintaining a therapeutic relationship [15].

Nurses are the staff members who have the most interactions with patients throughout the day [16]. Indeed, nurses represent the largest number of people working in mental health units [17]. Therefore, what happens in the interactions must be described in the nursing documentation of the clinical history [17]. Evidence suggests that the nursing records can be an effective way to improve nursing practice [18]. However, it has been shown that the quality of written mental health nursing documentation needs improvement to ensure continuity and patient safety [19]. Many nursing interventions that take place in the context of the therapeutic relationship are often not recorded in clinical courses [20], but they are more focused on problems and symptoms [21]. In light of the above, it is evident that there is a need for instruments to evaluate the quality of nurses’ clinical notes, especially in the context of records in which clinical information obtained in nurse-patient interactions is collected.

In order to assess the quantity and quality of interactions between nurses and patients described in clinical notes in mental health units, Myklebust and Bjørkly [2] developed and reliability tested the Scale for the Evaluation of Staff-Patient Interactions in Progress Notes (SESPI). This scale enables the evaluation of nurses’ clinical notes in relation to the quality of the patients’ experience, the approaches taken by the nurses and whether this approach meets the emotional needs of the patients [2].

Supporting the need and relevance of having an instrument to assess the quality of nursing clinical notes in acute mental health services in Spain, and given the usefulness of this instrument for assessing clinical notes in the original context, and whereas that no other tools have been found that assess the quality of nursing clinical notes and are suitable for acute mental health units, the aim of this study was to perform the cross-cultural adaptation of the SESPI scale to Spanish and to study its psychometric properties.

## Methods

### Design

**Fig 1:**
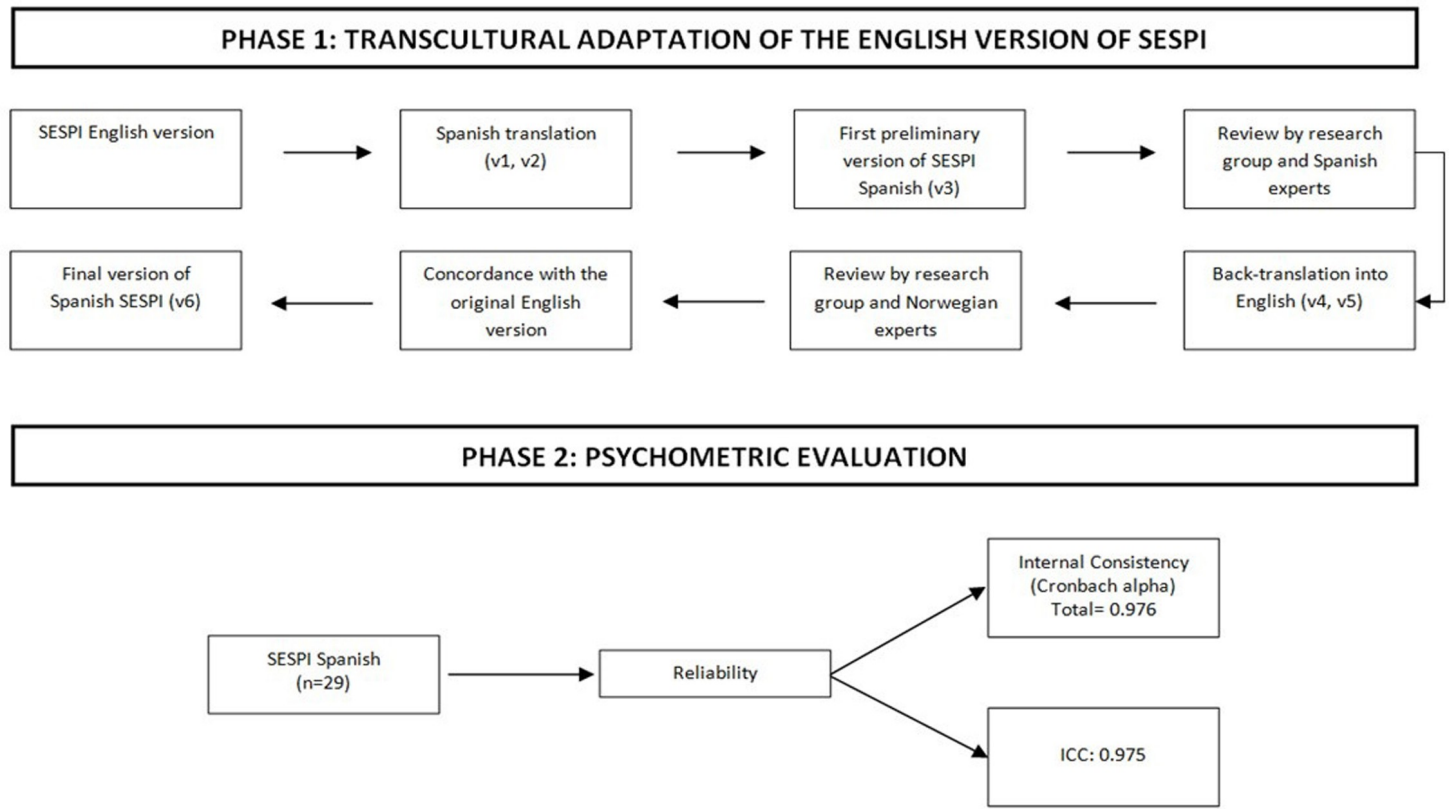
Overview of the two-phase study.

A psychometric study of cross-cultural adaptation and validation of the SESPI Scale into Spanish was performed. The study was conducted in two phases: in the first phase, the SESPI scale was adapted to Spanish and in the second phase the psychometric properties of the Spanish version were analysed (Fig 1).

### Instrument

The SESPI scale assesses the quality of the patient experience, the approaches taken by nurses and whether this approach meets the emotional needs of the patients [2]. This scale consists of the following four steps:

1. Step 1 aims to know whether the patient’s experience is described in the clinical note. It is answered dichotomously (Yes/No), and only an affirmative answer enables one to proceed to the next step.
2. Step 2 evaluates whether the patient’s experience in the interaction with the nurse has been positive or negative. Depending on the degree of satisfaction or dissatisfaction of the patient’s response, the answer is -II, -I, +I, +II.
3. Step 3 identifies and categorizes the nurse’s response to the interaction described in the clinical note. There are four possible responses: a) The nurse’s response is not described in the clinical note; b) The nurse’s response is known, but not the patient’s reaction; c) We know the nurse’s response and the patient’s reaction, but not the patient’s feelings; d) The nurse’s response, the patient’s reaction and the feelings produced by this intervention are noted. Only this response enables one to move on to the next step where the clinical note is evaluated regarding to attunement.
4. In step 4 this attunement is evaluated positively or negatively according to whether it was unsuccessful or successful. Again, the response is -II, -I, +I, +II depending on how unsuccessful or successful the attunement was.

Regarding the psychometric results of the original scale, the Cronbach’s alpha for the entire instrument was 0.977 and the ICC was 0.770 [2].

### Phase 1 procedure

The process of cultural adaptation of the Spanish version of the SESPI scale was carried out following the Standards for Educational and Psychological Testing [22]. Before beginning the translation, permission was requested from the authors for its adaptation to Spanish. The English version of the instrument was translated into Spanish by two translators, whose mother tongue was Spanish and who were fully fluent in English, providing two versions of the SESPI instrument (v1 and v2), which were synthesized to a single version (v3) by a committee of experts formed by four nurses specialized in mental health and a nurse specialized in psychometrics. This version was sent to two new translators who did not know the original version, whose mother tongue was English and who were fluent in Spanish, so that they could carry out the back-translation into English. The two versions obtained (v4 and v5) were compared with the original SESPI by the same committee of experts, resulting in version 6, which was revised together with the original authors.

A pre-test was then carried out on a sample of 19 nurses with experience in recording information in clinical records. To assess the comprehension and clarity of the items, as well as the format and time required to complete the instrument, four clinical notes were provided. The clinical notes that were selected were written by nurses in which the patient’s experience was present in the situation described and which narrated episodes in which the staff and patients had the opportunity to interact.

### Phase 2 procedure

For the reliability testing of the psychometric properties of the Spanish version of the SESPI-Sp instrument, 12 clinical notes were purposively selected from patient records from three different hospitals, representing the totality of possible responses to the scale. The clinical notes were collected in a single coded and anonymized document (S1 File). The selected clinical notes tried to represent the totality of the possible responses contained in SESPI, therefore, a heterogeneous sample was used trying to fulfil that purpose.

To facilitate data collection, an electronic adaptation of the SESPI scale was developed using the Microsoft forms platform. Thus, the research team sent each participating nurse an e-mail with the evaluator code, the document with the clinical notes to be evaluated, and the link to the form containing the SESPI scale, to be completed as many times as there were clinical notes to be evaluated.

### Participants

Following the recommendations by Bujang and Baharum [23], for the 12 observations (clinical notes) that had to be analysed by the evaluators in our study (statistical power of 80%, alpha = 0.05 and an effect size difference of ICC = 0.20, 20 nurses were required. Similarly, the criterion used in the original SESPI reliability test was also taken into account [2] accepting a detection of an effect size difference of 0.20 (ICC = 0.70 vs. 0.50) as being acceptable for testing instrument development.

The invitation and selection of the nurse evaluators were made according to the following criteria: nurses from the mental health hospitalization area, with experience in collecting and writing information in medical records, with at least two years of experience in mental health nursing. The nurses were selected by purposive sampling. Finally, 29 nurses participated.

### Statistical analysis

This study used the same statistical analysis paradigm for testing reliability as was used for the original SESPI [2]. Two tests were used to analyse the reliability of the instrument. The Kuder-Richardson 20 test was used for the dichotomous scale in step 1. The Cronbach’s alpha test was used to estimate the internal consistency of the scores for the total SESPI scale and for steps 2, 3 and 4. Likewise, the intraclass correlation coefficient (ICC) was chosen to calculate the inter-rater reliability of the distribution of scores for the 12 excerpts across the four steps of coding and for steps 1, 2, 3 and 4 separately. Statistical analyses were conducted using the Statistical Package for Social Science (SPSS) Version 27.

### Ethical considerations

The study was approved by the IRB Bioethics Committee (2021/9835) and the other participating hospitals. The authorization and participation of the authors of the original version of the instrument was requested for its cross-cultural adaptation. All participants signed the informed consent form. To maintain the anonymity of participants, the principal investigator previously assigned an evaluator code to each nurse, sent via e-mail along with the form using the Microsoft forms platform in which they entered their evaluations of the SESPI-Sp.

## Results

### Phase 1: Adaptation of the SESPI scale

The translation process was carried out systematically and involved discussions of semantic, idiomatic, and conceptual equivalence at each stage. Regarding the content of the instrument, most of the steps, categories and examples were simple and therefore easy to translate. Only a few words required adaptation to bring the meaning closer to the Spanish context in which SESPI was to be used. The translations of these words in *“v*.*1 and v*.*2”* were easily agreed upon by the expert committee. However, the most complex adaptation had to do with the term "excerpts" since it has several accepted translations in Spanish, but in the context of acute hospitalization units in Spain, "*nota del curso clínico*" or "*registro*" is normally used to define an "extract" of a clinical note.

**Fig 2:**
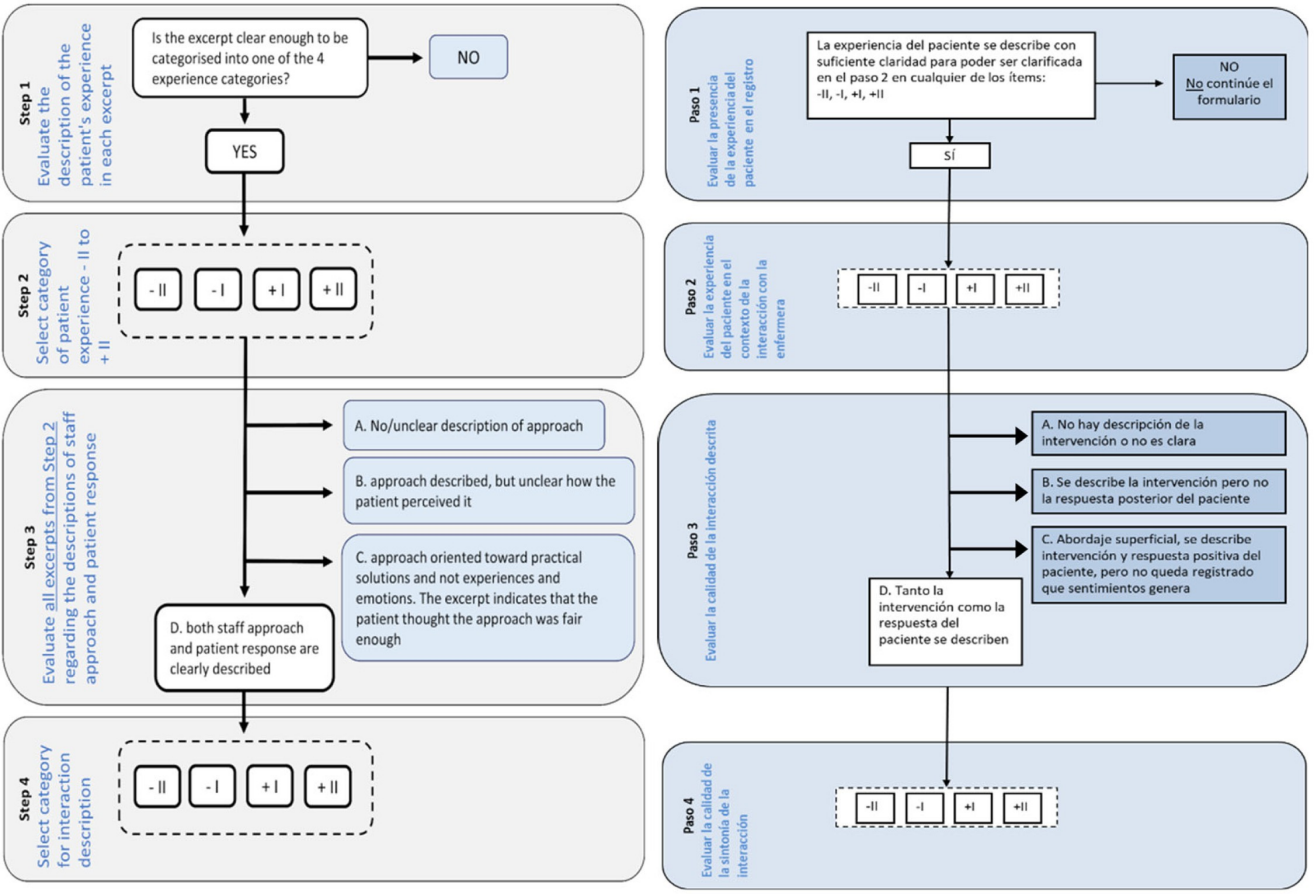
Structure of the original SESPI scale (Scale for the Evaluation of Staff Patient Interactions in Progress Notes) &SESPI-Sp].

Before finalizing the Spanish version of SESPI, a pilot test was performed in which 19 nurses participated and evaluated four clinical notes. The nurses who participated in the pre-test of the SESPI-Sp required an estimated average time to evaluate the four notes that ranged from 5 to 10 minutes and confirmed that it was easy to complete. After completing the SESPI-Sp, participants were asked to report any items with an unclear meaning. All such reported comprehension problems were discussed by three authors (APT, AMP, JRM) and used to inform the modifications of the SESPI-Sp. One of the most common comments was that the format and wording did not help to understand the steps. In this sense, the format of the tool was partially modified in an attempt to clarify how to correctly complete and use the resources made available, keeping the content and structure intact so as not to detract from the semantic essence. Thus, on the 1st and 2nd pages of the SESPI-Sp, instructions on how to respond to each of the four steps and how to categorize the responses were incorporated. Another slight modification to the format was to unify each of the categories belonging to the four SESPI steps in a single table. The purpose of this modification was to achieve a more intuitive view of the steps and categories and thus reduce the confusion generated in the pre-test. In the table for each category, a brief explanation of the interpretation of this step was included, which in the original SESPI is included before the examples of situations. For each of the steps, the original SESPI presents examples that help the user of the tool to identify the corresponding category according to what is written in the clinical note. These examples are written after the brief explanation of each of the step categories. The cross-culturally adapted version places these examples at the end of the tool to have them available for consultation in case of doubt, yet separate from the categorization section, to avoid distracting the user. These modifications to the format do not change the meaning or usefulness of the tool. The original authors were aware of these modifications and gave their approval. The suggestions and proposals for improvement that they provided in their feedback were considered and were included in the final version, which was named SESPI-Sp (S2 File). Fig 2 shows the structure of SESPI and SESPI-Sp.

### Phase 2: Psychometric properties of the SESPI-Sp

#### Participant characteristics

Table 1 shows the characteristics of the 29 participating nurses who served as evaluators in the study. The age of the nurses ranged from 26 to 59 years, with a mean age of 35.6 years (SD = 8.7). Almost 60% of the nurses were female. Their experience in mental health was a mean of 10.9 years (SD = 9.1). More than half had the official title of mental health nurse specialist and had a doctoral or master’s degree. Their experience writing clinical notes ranged from 3 to 38 years, with a mean of 10.6 years (SD = 8.5).

**Table 1:** Participants’ sociodemographic and professional characteristics (n = 29)].

Variable	n (%)
Mean age, years ± SD	35.6 ± 8.7
Gender	
Male	12 (41.4%)
Female	17 (58.6%)
MH nursing specialty	
Yes	15 (51.7%)
No	14 (48.3%)
Highest education	
Bachelor’s degree	13 (44.8%)
PhD or master’s degree	16 (55.2%)
Mean MH experience, years ± SD	10.9 ± 9.1
Mean experience writing clinical notes, years ± SD	10.6 ± 8.5

Data are presented as number (percentage) or means ± standard deviation (SD).

MH, mental health.

#### Inter-rater and internal consistency reliability of SESPI estimated for the entire instrument

The Cronbach’s alpha for the total instrument was 0.976. As in the original version, this value indicates that the raters’ scores had a very high consistency for the four steps of the SESPI.

#### Inter-rater and internal consistency reliability for each step of the SESPI

Step 1 was completed using a dichotomous yes or no scale. The reliability analyzed using the Kuder-Richardson-20 coefficient was 0.949. The ICC for step 1 was 0.942 (95% CI: 0.082–0.995).

Steps 2 to 4 were evaluated using a four-point ordinal scale. Table 2 shows the values of Cronbach’s alpha and the intraclass correlation coefficient for steps 2, 3 and 4. Values above 0.941 were obtained for Cronbach’s alpha and the ICC for the total and for each of the steps analysed.

**Table 2:** Internal consistency and ICC for the last three steps and total of the SESPI-Sp.

SESPI-Sp Steps	Cronbach’s alpha	ICC	95%CI
Step 2 n = 29	0.944	0.941	0.881–0.980
Step 3 n = 29	0.986	0.986	0.971–0.995
Step 4 n = 29	0.944	0.948	0.895–0.982
SESPI-Sptotal	0.976	0.975	0.962–0.985

SESPI-Sp:SESPI Spanish version, ICC: the intraclass correlation coefficient, CI: confidence interval.

## Discussion

This study aimed to carry out a cross-cultural adaptation and test the reliability of the Spanish version of the SESPI instrument. The purpose of this instrument is to evaluate the quality of nurse-patient interactions recorded in clinical notes in mental health hospitalization services.

Regarding the adaptation process, it should be noted that it was carried out swiftly and here were hardly any discrepancies among the participants during both the translation and back-translation processes; only some terms required adaptation to the Spanish context. This fact confirms the equivalence in meaning and the reliability of the semantic content of the Spanish version of the instrument [24]. Another important aspect that should be emphasized is that during the adaptation process the structure of the tool required a slight modification in terms of where the indications and examples were provided, although this did not lead to changes in the sense and meaning of the content of the instrument. The fact that this was the result of the recommendations of the participating clinical nurses, that the expert committee evaluated it positively and, moreover, that it received the approval of the original authors, guarantees the level of reliability of the adapted version of the instrument [24, 25].

In relation to the psychometric results, it should be noted that the reliability of the Spanish version of the SESPI showed excellent results regarding internal consistency and interobserver reliability. In the case of the total Cronbach’s alpha test it was 0.976, a very similar result to that of the original study testing the SESPI’s reliability [2]. In the case of the ICC, the result for the total scale was 0.975, indicating very good interobserver reliability [26]. In fact, for some authors, above 0.81 is considered almost perfect [27]. This result was substantially higher than that obtained in the original SESPI version [2], this finding could be due to a larger number of observations and observers in this study. For an in-depth study of the results of the different stages that could be answered with the instrument, 12 clinical notes were selected that included the different options that could be presented and were evaluated by 29 nurses with different profiles of clinical experience in mental health. In this sense, the results obtained for each stage of the instrument were also very acceptable, obtaining Cronbach’s alpha values above 0.94 for all the steps, along the same lines as the original version [2].

### Strengths and limitations

Regarding the strengths of the study, in the first place, standardized translation and cross-cultural adaptation procedures were used [22]. SESPI has the great advantage of being a short questionnaire that requires only a short time for evaluation. This study reviewed numerous clinical notes from acute mental health units in several hospitals in Spain. However, for the psychometric study, 12 clinical notes were selected, in order to represent the different possible responses to the SESPI and to study the consistency of the responses in detail. The lack of scales for evaluating the quality of the clinical notes translated and validated in Spanish did not prevent us from reproducing the process of evaluating the psychometric properties so that it would be similar to that of the original study. The participants were recruited from a group of nurses in the mental health field and the selection process considered that the profiles of the nurses were representative of the group. However, the nurses who acted as evaluators showed a high education level and motivation to collaborate in this study. This fact could have conditioned the results. The findings may not be directly generalisable to nurses and staff with different educational backgrounds. A scale’s reliability is not a fixed quality, but depends on the specific group of raters and their training in the use of the scale [28]. Clearly, when the SESPI is used in research projects, new reliability tests are required for each project. This does not mean that our reliability testing of the SEPSI is superfluous. Any development of a new test or instrument requires reliability and validity testing. This is the first study measuring clinical nursing notes using the Spanish version of the SESPI-Sp instrument, new studies are required, with larger samples, to validate our findings.

### Implications for practice

The process of translation, cross-cultural adaptation, and evaluation of psychometric properties indicated that SESPI-Sp is an instrument capable of providing valid and reliable measures to assess the quality of clinical notes in acute mental health inpatient services in the Spanish context. Therefore, this study has special relevance for management, clinical practice, teaching and research. With this tool, a critical examination of nursing documentation and the identification of gaps in nursing writing is achieved. Firstly, its use allows managers to be aware of the quality of the records. In addition, it is relevant for clinical practice, as being able to properly record interventions following specific steps, nurses will be more aware of the steps they need to follow to correctly record patients’ progress notes. In this sense, it can be an important tool for nurses’ decision-making processes, especially for planning goals and interventions in the context of patient interactions. In addition, knowing how to critically evaluate records can generate further knowledge related to the quality of the nursing record in the patient’s clinical notes. The availability of this instrument may be useful for designing training programs for nurses oriented towards the problem areas described by nurses and confirmed by patients’ expectations. Finally, new lines of research are opened that may generate further knowledge related to the quantity and quality of the nursing record in the patient’s clinical notes.

## Conclusions

This study has enabled a Spanish adaptation of an instrument to assess the quality of nurse-patient interactions recorded in clinical notes, and to study its psychometric properties. The SESPI-Sp is a simple, easy to administer and reliable tool for assessing aspects of the quality of mental health care. The results of the reliability tests performed with the SESPI-Sp confirm its usefulness for quantitative research as was the case with the original tool. The SESPI can be used to measure the quantity and quality of nurse-patient interactions described in nurses’ clinical notes. The SESPI-Sp measures patient interactions from the perspective of attunement, including the quantity and quality of patient experiences described, staff approaches, and whether the approaches succeeded in meeting patients’ needs. The SESPI-Sp can provide data to evaluate nursing documentation qualitatively in relation to nursing interventions delivered in the context of interactions with patients in mental health units. Thus, contributing to the improvement of the quality of nursing care in mental health.

## Supporting information

S1 File Progress notes analysed. (DOCX)

S2 File Scale for the Evaluation of Staff Patient Interactions in Progress Notes. (PDF)

S3 File Study data. (XLSX)
