# Supplementary material for: Transcultural adaptation and assessment of psychometric properties of the Spanish version of the Scale for the Evaluation of Staff-Patient Interactions in Progress Notes
Source: PLoS One. 2023 Mar 28;18(3):e0281832. doi: 10.1371/journal.pone.0281832 (PMC10047521; doi:10.1371/journal.pone.0281832)
Supplement: S1 File — (DOCX) [file pone.0281832.s001.docx]

**PROGRESS NOTES**

**NCV1:** After lunch punctually labile and irritable due to expansive and invasive behavior of another user. She explains that today she is sadder because just this day her father died.

**NCV2:** She comes to the resopon, apparently calm and adequate. She asks to be rescued, saying that otherwise she will not be able to fall asleep. Conciliation insomnia.

**NCV3:** Expansive, irritable patient. Vociferous, refers to being angry with TN. Constant demands to smoke, we explain that it is not possible, we offer chewing gum and patches "I don't want any of that, I need to smoke", she looks for a doctor who is not a referral doctor, when she receives a refusal, she bangs on the door of the room, crying. We set limits that are poorly tolerated, then she screams and cries. She maintains delirious discourse of the bank.

**NCV4:** Adequate intake at dinner and snore. After dinner he is very insistent about wanting to talk to the doctor on call, I verbally redirect him because of his increasing irritability, he refers to having headache, after consulting with the doctor on call I give him ibuprofen v.o., he entertains himself playing board games with other patients, at the resopon he refers to feeling much better.

**NCV5:** According to the patient she has made a call to her partner that has gone bad. Explains fear of losing her family after finding out about her drug use. Wishes to be discharged. She is tearful.

**NCV6:** She collaborates in the taking of constants. She shares spaces and entertains herself playing board games, she still does not communicate verbally with the rest of the patients but does so by means of gestures. She receives a visit from her father, with no relevant incidents. Correct ingestions. *Close supervision during the shift.

**NCV7:** 21.30H, he is watching TV. When he sees the news about the volcano in La Palma, he comes to the control room banging. Verbalizes that we are killing the island, that we are... Nazis. He asks to speak to the police and politicians. We try to reconduct verbally, he starts to paint, takes a shower and seems to calm down, he does not want a rescue pattern.

**NCV8:** Active per room during the shift. Somewhat anxious about losing a card, we redirect and she is calmer.

**NCV9:** Her daughter comes to visit her, good contact during the visit but when she leaves she is labile and needs to be accompanied.

**NCV10:** Very insistent on giving her the medication at dinner because she used to take it that way, she is worried about this issue in case something happens to her. I talk to her, she is satisfied but it does not seem right to her.

**NCV11:** Refers that she is having a hard time not being able to see her daughter, although she understands that she has to be here to be okay when she gets out. Desires to stop using, which I value positively. She explains the frequency and pattern of use. I do active listening and emotional venting. She is tearful as we talk. After that she is given Quetiapine 25mg s/p.

**NCV12:** Refers that the weekend went very well, she values positively being
