# Supplementary material for: Transcultural adaptation and assessment of psychometric properties of the Spanish version of the Scale for the Evaluation of Staff-Patient Interactions in Progress Notes
Source: PLoS One. 2023 Mar 28;18(3):e0281832. doi: 10.1371/journal.pone.0281832 (PMC10047521; doi:10.1371/journal.pone.0281832)
Supplement: S2 File — (PDF) [file pone.0281832.s002.pdf]

**Scale for the Evaluation of Staff Patient Interactions in Progress Notes Spanish (SESPI-Sp)**

## PASO 1: Evaluar la presencia de la experiencia del paciente en el registro

**<u>Si</u>**

- La experiencia del paciente se describe con suficiente claridad como para poder clasificarla en el paso 2 en cualquiera de los ítems:
- \- I, -II, +I, +II .

**<u>No</u>**

- Ninguna descripción de la experiencia del paciente o descripción insuficiente para la categorización en el paso 2. *(ej: pasa el turno en la habitación / duerme durante toda la noche)*
- <u>No</u> continúe el formulario.

## PASO 2: Evaluar la experiencia del paciente en el contexto de la interacción con la enfermera

**- II <u>Muy incómodo</u>**

**El paciente expresa explícitamente sentimientos / pensamientos / experiencias muy incómodas o negativas.**

**Interacción que muestra que el paciente se siente muy incómodo** *(ej: ataca al personal, da un portazo)*

**La enfermera evalúa que el paciente se siente muy incómodo** sin ser confirmado por el paciente (valoración subjetiva) *(ej: parece estar muy deprimido).*

**– I <u>Incómodo</u>**

**El paciente expresa explícitamente sentimientos / pensamientos / experiencias incómodos**

**Interacción que muestra que el paciente se siente incómodo** *(ej: pide que lo dejen en paz. No responde a las preguntas que se le hacen)*

**La enfermera evalúa que el paciente se siente incómodo** sin ser confirmado por el paciente (valoración subjetiva) *(ej: parecía ansiosa)*

**+I <u>Positivo</u>**

**El paciente expresa explícitamente sentimientos / pensamientos / experiencias positivas**

**Interacción que demuestra que el paciente tiene una experiencia positiva**

**La enfermera <u>evalúa</u> que el paciente se siente mejor** sin ser confirmado por el paciente (valoración subjetiva). *(ej: parecía aliviado y más tranquilo después de que le dijeran que su estancia en la sala se mantenía unos días más).*

**+ II <u>Muy positivo</u>**

**El paciente expresa vitalidad, alegría o confianza en sus propios recursos**

**Interacción que muestra una experiencia positiva por parte del paciente**

**La enfermera evalúa que el paciente se siente muy feliz o está mejorando** (sin ser confirmado por el paciente (valoración subjetiva).

## PASO 3: Evaluar la calidad de la interacción descrita

**A** No hay descripción de la intervención de la enfermera o la descripción no es clara. (*ej: dice que el paciente ha estado ansioso, pero nada sobre lo que se hizo en respuesta*).

**No** completar el paso 4.

**B** Se describe la intervención, pero no describe la respuesta posterior del paciente (*ej: estaba preocupada y ansiosa y se la invitó a hablar sobre ello*).

➢**No** completar el paso 4.

**C** Abordaje superficial, se describe la intervención y una respuesta positiva o de aceptación del paciente, pero no queda registrado que sentimientos genera su respuesta. (*ej: - Estaba ansiosa y le ofrecieron una píldora ansiolítica que aceptó)*

➢**No** completar el paso 4.

**D** Tanto la intervención de la enfermera como la respuesta del paciente se describen de tal manera que se puede juzgar si el paciente se sintió satisfecho o no.

**Completar el paso 4**

## PASO 4: Evaluar la calidad de la sintonía en la interacción

**-II Sintonía Fallida**

**Intervención de la enfermera en la que no se muestran intentos de comprender al paciente (***ej: el profesional ordena que se vaya a su habitación sin querer escuchar más explicaciones***) Y**

**El paciente percibe claramente el enfoque como negativo.**

Registros en los que se describe que el personal usa una posición dominante o de experto y la respuesta del paciente es fuertemente negativa.

**– I Sintonía Parcialmente fallida**

**Intervención de la enfermera que impide una mayor interacción a pesar del hecho de que es más leve que - II Conexión fallida (incluso puede ser un intento de comprender al paciente) PERO/A PESAR DE ELLO**

**El paciente se retira o responde explícitamente negativamente al abordaje, pero en menor medida que en la categoría –II**

Extractos ligeramente autoritarios, el personal ha intentado comprender el estado del paciente, pero la reacción de este fue negativa.

**+I Sintonía parcialmente exitosa**

**Intervención de la enfermera que parece estar tratando de comprender / explorar la experiencia del paciente con palabras o acciones, pero el contenido de la intervención no se describe adecuadamente Y**

**El paciente percibe una experiencia positiva con respecto a esta intervención, o que la intervención hizo posible una mayor interacción positiva** *(ej: el paciente comenzó a explicar más sobre su situación)*

Registro que incluye la intervención del personal y la respuesta positiva del paciente, a pesar de que este no ha verbalizado expresamente esta emoción.

**+ II Sintonía exitosa**

**La intervención de la enfermera está claramente orientada a tratar de comprender / abordar / verificar la experiencia del paciente con palabras o acciones Y**

**El paciente percibe esta intervención como una experiencia positiva, o se abre claramente para una mayor interacción.**

Incluye acercamientos para explorar o confirmar la experiencia de un paciente y los comentarios positivos del paciente. Requiere descripciones exactas de la intervención de la enfermera.
